# A Spatial Framework to Map Heat Health Risks at Multiple Scales

**DOI:** 10.3390/ijerph121215046

**Published:** 2015-12-18

**Authors:** Hung Chak Ho, Anders Knudby, Wei Huang

**Affiliations:** 1 Department of Geography, Simon Fraser University, Burnaby, BC V5A 1S6, Canada; 2 Department of Geography, Earth and Environmental Sciences, Okanagan College, Kelowna, BC V1Y 4X8, Canada; 3 Department of Geography, University of Ottawa, ON, K1N 6N5, Canada; aknudby@uottawa.ca; 4 Department of Geography, University of Wisconsin-Milwaukee, Milwaukee, WI 53201, USA; weihuang@uwm.edu

**Keywords:** heat risk, modifiable areal unit problem, heat vulnerability, extremely hot weather event

## Abstract

In the last few decades extreme heat events have led to substantial excess mortality, most dramatically in Central Europe in 2003, in Russia in 2010, and even in typically cool locations such as Vancouver, Canada, in 2009. Heat-related morbidity and mortality is expected to increase over the coming centuries as the result of climate-driven global increases in the severity and frequency of extreme heat events. Spatial information on heat exposure and population vulnerability may be combined to map the areas of highest risk and focus mitigation efforts there. However, a mismatch in spatial resolution between heat exposure and vulnerability data can cause spatial scale issues such as the Modifiable Areal Unit Problem (MAUP). We used a raster-based model to integrate heat exposure and vulnerability data in a multi-criteria decision analysis, and compared it to the traditional vector-based model. We then used the Getis-Ord G_i_ index to generate spatially smoothed heat risk hotspot maps from fine to coarse spatial scales. The raster-based model allowed production of maps at spatial resolution, more description of local-scale heat risk variability, and identification of heat-risk areas not identified with the vector-based approach. Spatial smoothing with the Getis-Ord G_i_ index produced heat risk hotspots from local to regional spatial scale. The approach is a framework for reducing spatial scale issues in future heat risk mapping, and for identifying heat risk hotspots at spatial scales ranging from the block-level to the municipality level.

## 1. Introduction

Climate change is influencing the severity and frequency of heat waves [1,2], which may lead to increasing heat-related morbidity (e.g., cardiovascular and respiratory diseases) and mortality, especially during extreme heat events [3]. Excess mortality from extreme heat is a worldwide phenomenon, occurring in the tropics [4], subtropics [5], and temperate climate zones, the latter ranging from the extreme heat event in Central Europe in 2003 [6,7] to cooler places such as Vancouver, Canada, in 2009 [8]. The health effects of extreme heat are influenced by the severity and duration of the extreme heat event, compounded by simultaneous effects of air pollution as well as population vulnerability [3], and modified by typical summer temperatures to which the population is adapted [9,10]. In order to address related public health impacts, previous studies have temporally evaluated a range of temperature metrics to estimate heat-related mortality [11,12], have estimated spatial and temporal variability in heat-related mortality [13,14,15,16,17,18,19,20], and have developed indices to locate heat vulnerable populations [2,21,22,23].

**Figure 1:**
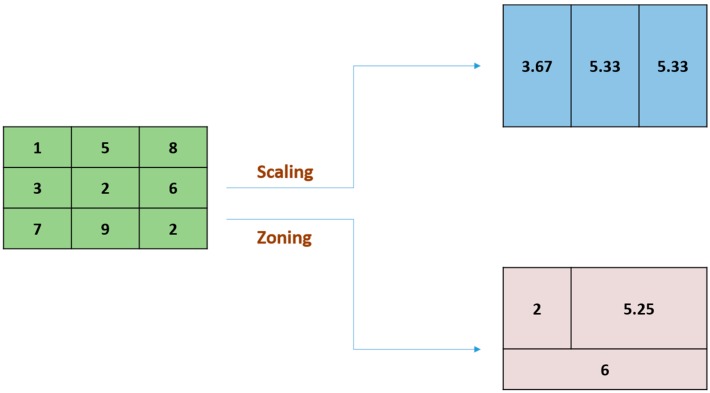
An illustration of the Modifiable Areal Unit Problem (MAUP). On the left side an original high-resolution dataset is shown, while the right side illustrates two different results of spatial aggregation of the original data from scaling (scaled to coarser scale/resolution with same spatial size) and from zoning (scaled to the coarser scale/resolution by areas with different spatial sizes). The spatial pattern of the averaged values depends on the boundaries of the spatial units of aggregation.

Although previous studies have quantified and mapped population heat vulnerability, only a few studies have combined heat vulnerability information with data on heat exposure to evaluate the health risks associated with extreme heat [24,25,26]. Identification of heat risk hot spots can be used to guide heat mitigation interventions, such as establishment of green or reflective roofs, urban parks, water features, *etc.* Furthermore, most heat health risk studies have aggregated data to match spatial units employed by the census from which vulnerability information is typically derived. However, the choice of the spatial unit used to calculate vulnerability (e.g., postal code, census tract, *etc.*) affects the identification of vulnerably neighborhoods [23], and introduces potential spatial data quality concerns due to the relatively coarse spatial resolution of census information and the modifiable areal unit problem (MAUP), a statistical bias that arises from the selection of a specific spatial unit of analysis [27,28,29,30]. When geographic data are aggregated into a spatial feature with specific spatial scale (e.g., by census tract, postal code, or county), mean values within the spatial feature are affected by its boundary (Figure 1), which in turn may lead to a zoning effect that affects subsequent analyses. For example, if a mean temperature value is assigned to a census tract that temperature will be lower if the census tract includes a local lake than if the boundary had been drawn such that the lake belonged to the neighbouring census tract, although the temperature that the population is exposed obviously remains unaffected by the administrative boundary. This can lead to substantial errors when temperature maps are used as proxies for the heat exposure of the population living in each census tract, especially because temperature is strongly influenced by the local landscape [31]. According to results by Sobrino *et al.* [32] the optimum spatial resolution of surface temperature maps could be as low as 50 m when mapping the district-level surface urban heat island and 100 m is sufficient to describe differences between neighborhoods, while a spatial resolution of 500 m or coarser does not allow a description of significant temperature differences between neighborhoods. Spatial units commonly used to quantify population vulnerability, such as dissemination areas, census tracts and counties, are thus very coarse (typically > 1 km) compared to the local variability in temperature found in urban environments. An even greater problem exists when a local weather station is assumed representative of a study area that extends beyond its neighbourhood [3]. In such cases spatial interpolation may be used to estimate neighbourhood-level temperatures between weather stations, but inadequate coverage, uneven distribution of weather stations, and the influence of the local landscape on temperature typically render such approaches suboptimal [33].

The objectives of this study were: (1) to develop an alternative raster-based spatial framework to estimate heat health risk at high spatial resolution; (2) to examine the differences between the resulting raster-based map and a comparable map produced with the traditional vector-based approach; and (3) to predict and map the heat risk hotspots at multiple spatial scales for our study area, the greater Vancouver area, British Columbia, Canada.

## 2. Methods

### 2.1. Study Site

**Figure 2:**
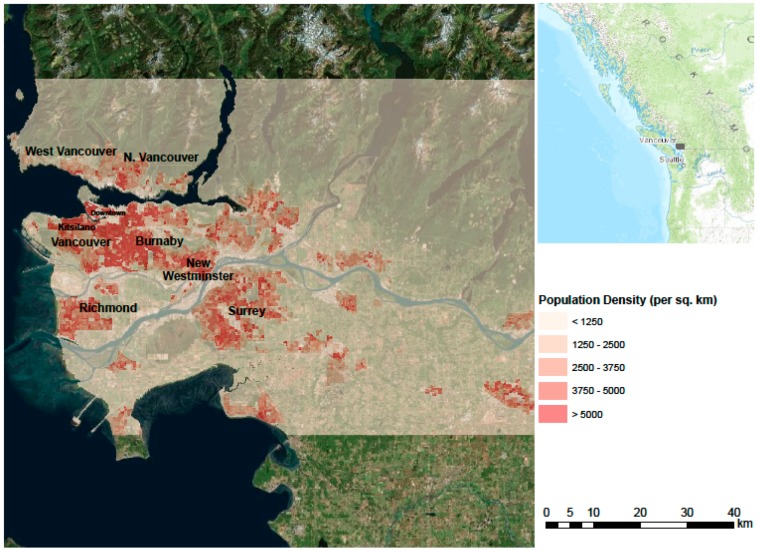
Study site in the greater Vancouver area. A Landsat 5 TM image forms the background, the extent of the study area is indicated in semi-transparent grey, and the population density is shown in shades of red.

The greater Vancouver area is a coastal metropolis with a population of more than two million people [34] and a moderate, temperate climate (Figure 2). During the summer of 2009 the greater Vancouver area experienced an unprecedented extreme heat event [15] that was associated with more than 100 excess deaths over a 7-day period [8]. It was the first clear indication that high ambient temperatures could adversely affect the population of greater Vancouver. Immigration and rapid development has created an environment in greater Vancouver with a high percentage of the population living alone and in multi-storey buildings, two factors known to increase heat health vulnerability [35,36]. According to the 2006 census [34], 28.46% of the population in the Greater Vancouver Census Division live in single-person households, and 39.41% live in the multi-storey apartments. This includes 12.76% living in high-rise buildings with more than five storeys. Furthermore, as elsewhere in Canada and beyond, the area is experiencing a gradual aging of the population [34].

### 2.2. Data

#### 2.2.1. Vulnerability Data

Despite geographical variation, population groups at elevated mortality risk during extreme heat events have typically been found to include: (1) seniors; (2) infants; (3) people living in old housing, multi-story apartment buildings, and mobiles homes; (4) people living alone; (5) people with low income; (6) people with low education; and (7) the unemployed. Seniors are widely recognized as a heat vulnerable population [25,26,37,38]. Seniors typically exhibit lower tolerance of extreme heat due to comorbidities, reduced mobility, reduced ability to care for themselves, and weaker immune systems [39,40], which increases their susceptibility to heat-related morbidity and mortality during extreme hot weather events [7,41]. Infants are primarily at risk due to low self-awareness of dehydration combined with inability to cool themselves, and rely on parents for cooling [42]. Heat has specifically been shown to be a factor in Sudden Infant Death Syndrome [43], and some suggest that the heat health risk of infants is usually underestimated [44]. People who live in older housing can be at risk because many older buildings do not have air conditioning and rely on natural ventilation using open windows and doors during hot weather [36]. This can result in heat trapped indoors and thus increased heat exposure, especially during stable atmospheric conditions with little wind [45], conditions that are typical of extreme heat events in the area [46]. Similarly, living in the upper level of a high-rise building or in a mobile home is associated with greater heat-health risk [25,36,45]. People living alone have increased risk of social isolation, which reduces their ability to seek and receive care when needed [36,45]. People with low income are at elevated risk as they have fewer resources for coping with extreme heat and, on average, also suffer more from comorbidities. As an example at the extreme end of the poverty spectrum, the homeless population in greater Vancouver has elevated incidence of mental illness as well as an elevated incidence of health problems stemming from drug use and alcohol consumption [47,48,49]. This population group has been shown to be at particular risk of heat stroke [50]. People with a lower education level are at elevated risk because they are more likely to do physically demanding work outdoors, which increases their heat exposure [51]. Finally, while unemployment in itself may not lead to increased risk, it can be associated with both social isolation and low income, which in turn may lead to poor living conditions. The unemployed have been demonstrated to be at elevated mortality risk during extreme heat events [36,50,52]. Acclimatization to heat can reduce the effect of these vulnerabilities on morbidity and mortality [9,10].

It is important to note that not all these variables have been shown to influence heat health risk in the greater Vancouver area [8,15], and that the climatic, social and infrastructural context is likely to modify their local importance. However, until more evidence of specific locally important heat vulnerability variables emerges, these variables are a reasonable list of factors likely to influence heat health vulnerability in the area. Data on these variables were extracted from the 2006 Canadian Census at the dissemination area level, a spatial unit that contains an approximate population of 400–700 persons, using the SimplyMap 3.0 database (Table 1). The physical size of dissemination areas in British Columbia varies between 839 m^2^ and 121,589 km^2^. Apart from the unemployment rate, all values were divided by the physical size of the dissemination area to obtain density values.

**Table 1:** Social vulnerability variables used to quantify heat vulnerability.

Variables Name	Details
Seniors	Number of people more than 55 years old
Infants	Number of people less than 5 years old
People in old houses	Number of households living in housing built prior to 1970
People in high heat risk homes	Number of households living in multi-story apartment buildings or mobile homes
Low income population	Number of people with annual household income less than $20,000
Low education population	Number of people without a diploma or a degree
People living alone	Number of single-person households
Unemployment	Unemployment rate

#### 2.2.2. Heat Exposure Data

Heat exposure was estimated using the land surface temperature (LST) derived from a Landsat 5 TM image from 23 July 2006, covering the full study area, resampled to 60 m spatial resolution. The date the image was acquired was a typical hot summer day in the study area, with a maximum temperature of 26.7 °C at Vancouver International Airport, light wind and no clouds. LST was estimated from Landsat TM band 6 based on the LST calculation from Ho *et al.* [31]. To obtain LST, we calculated the blackbody radiance at temperature LST based on the upwelling atmospheric radiance ($L^{↑}$), downwelling atmospheric radiance ($L^{↓}$) and atmospheric transmittance τ from NASA’s Atmospheric Correction Parameter Calculator [53], and the emissivity values (ԑ) from the North American ASTER Land Surface Emissivity Database [54], with the following equation:

(1) $$B(LST)=\frac{L_{sen}-L^{↑}}{\varepsilon \tau }-\frac{1-\varepsilon }{\varepsilon }L^{↓}$$

where B(LST) is the blackbody radiance at temperature LST and L_sen_ is the at-sensor radiance [55]. After that, an inversion of Planck’s Law was applied with the B(LST) to obtain the LST:

(2) $$LST=\frac{K_{2}}{\ln(\frac{K_{1}}{B\left(LST\right)}+1)}$$

where K1 and K2 are the thermal band calibration constants.

### 2.3. Multi-Criteria Decision Analysis

We used multi-criteria decision analysis (MCA) with two different data resampling approaches to visualize the influence of the MAUP issue on heat health risk maps. MCA is a qualitative statistical method that allows users to combine data layers in an analysis by assigning weights that represent the importance of each layer. Weights are typically based on expert knowledge. MCA proceeds by first assigning weights to each variable, then calculating a per-cell weighted average, and (optionally) discretizing the result based on percentiles or natural breaks. The method has been widely applied to risk analysis, for example in predicting landslide susceptibility, sinkhole mapping, soil erosion, and the potential for land development [56,57], as well as in the spatial heat-health literature [2,24,25,26]. We used MCA to map the heat-risk hotspots by combining heat vulnerability and exposure data layers in an application of Crichton’s Risk Triangle [58]. The eight individual heat vulnerability layers were classified using Jenks natural breaks [59], resulting in eight new vulnerability layers each with an index value from 1 (lowest vulnerability) to 9 (highest vulnerability). These eight layers were then combined into a single composite heat vulnerability layer by assigning equal weights (12.5%) to each layer, and the composite layer was reclassified using Jenks natural breaks in order to stretch the weighted values to a range of 1 to 9. The heat exposure layer was similarly classified using Jenks natural breaks, resulting in a layer with index values ranging from 1 (coolest, lowest exposure) to 9 (highest exposure, warmest). The composite vulnerability and exposure layers were then combined into a heat risk layer by assigning equal weights (50%) to each, and reclassifying the result into 9 new index values ranging from 1, indicating lowest risk resulting from a combination of low vulnerability and low exposure, to 9, indicating highest risk resulting from a combination of high vulnerability and high exposure.

**Figure 3:**
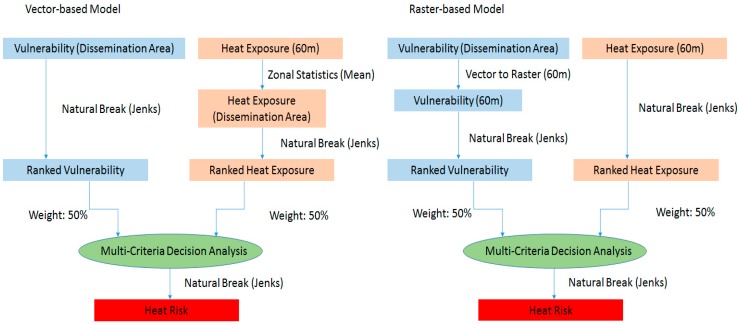
Flow Diagram from vector-based analysis (**Left**) and vector-based prediction (**Right**).

The vulnerability and exposure data layers were combined using two different resampling approaches. In the raster-based approach, the spatial structure of the heat exposure data (raster format with 60 m cell size) formed the basis of the overlay analysis. The vulnerability data layers were resampled to this format by extracting, for each cell, the value from the dissemination area covering the cell center. In the vector-based approach, the spatial structure of the heat vulnerability data (vector format defined by the census dissemination areas) was retained, and heat exposure values for each dissemination area were calculated as the mean of all cells within each vector polygon. We then compared the heat risk maps resulting from these two approaches to investigate differences in the resulting spatial patterns (Figure 3).

### 2.4. Getis-Ord G_i_ Index

Subsequently, we used the Getis-Ord G_i_ index [60] to improve the visualization of hotspots of heat risk at varying scales and reduce the zoning effect caused by the MAUP problem. The G_i_ index is a spatial statistic that identifies clusters of high and low values in a spatial data set by comparing values in a neighbourhood to the distribution of values in the complete data set. G_i_ is calculated as:

(3) $$G_{i}(d)=\frac{\sum_{j}w_{ij}(d)x_{j}}{\sum_{j}x_{j}}(j\mathrm{not}\mathrm{equal}\mathrm{to}i)$$

where d is the distance between cells, $w_{ij}\left(d\right)$ is a symmetric one/zero spatial weight matrix with ones for all links defined as being within d of a given i, and zero are the all other links including the link of point i to itself.

The G_i_ index quantifies the z-score of a cluster of values defined using a spatial lag distance [61]. Higher G_i_ values indicate a high-value cluster, *i.e.*, a hotspot, while lower values indicate a cold spot. Lag distances between one and four pixels, corresponding to approximately 200 and 500 m radii around each cell, were used to define the clusters and assess the effect of varying lag distance.

## 3. Results

### 3.1. Raster-Based and Vector-Based Heat Risk Maps

**Figure 4:**
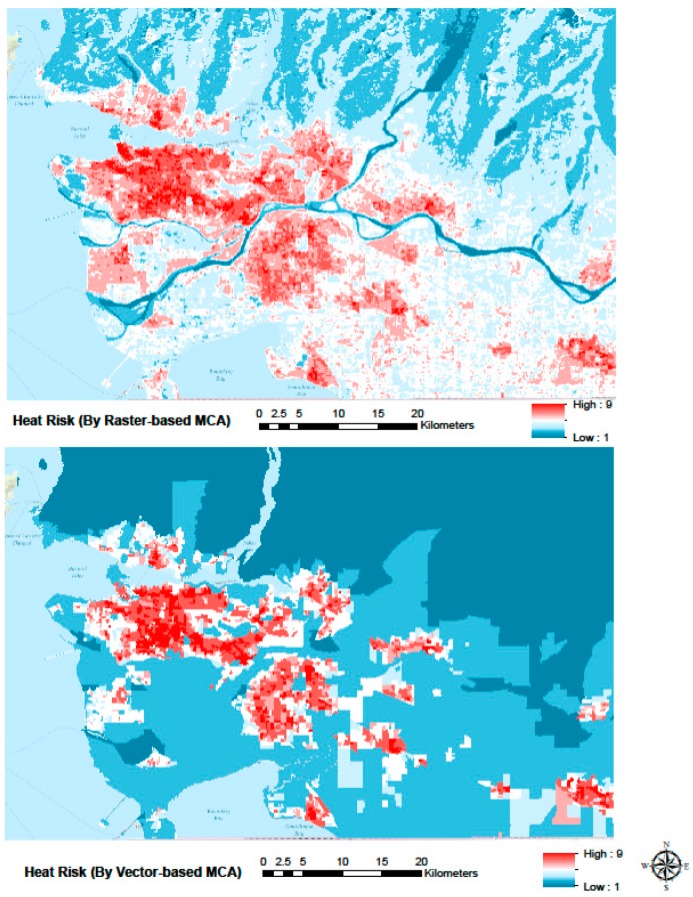
Heat risk maps from raster-based analysis (**Top**) and vector-based prediction (**Bottom**). Red indicates relatively high heat health risk, blue indicates relatively low heat health risk.

Heat health risk maps produced for the greater Vancouver area, using the two resampling approaches and the census and satellite data from 2006, are shown in Figure 4.

**Figure 5:**
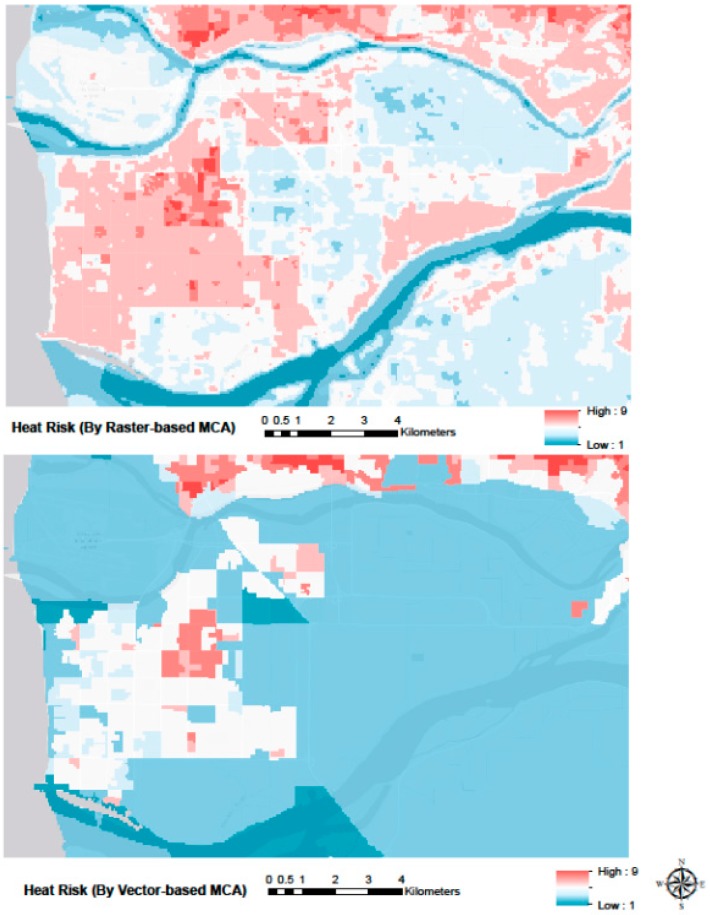
Heat risk maps from the raster-based (**Top**) and vector-based (**Bottom**) approaches zoomed into the city of Richmond. There is substantial discrepancy between the two approaches in the classification of heat health risk for this area.

The broad spatial patterns of heat health risk are similar between the two approaches, and indicate high risk in areas with a concentration of people with known vulnerability factors such as in the areas dominated by high-rise buildings (e.g., downtown Vancouver, and New Westminster), areas with relatively low income (e.g., South Burnaby and East Vancouver), and areas with a substantial immigrant population [62]. Areas known to be relatively hot, such as the extensive low-medium density neighbourhoods in Vancouver, Burnaby, Surrey and Richmond are also described as having relatively high risk. Within this broad spatial pattern, the maps also outlined several instances of local variability in heat health risk, such as in Burnaby, Richmond, and in the east end of downtown Vancouver, where substantial neighbourhood-level differences in vulnerability and exposure exist. The raster-based approach typically allowed description of greater local-scale variability than the vector-based approach, which largely classified the densely populated regions of Kitsilano, East Vancouver, Central Burnaby and New Westminster with a single index value. In addition, the raster-based and vector-based approaches typically portrayed local variability differently, as illustrated for Richmond in Figure 5. While the most densely populated part of Richmond is identified as a moderate hot spot by both approaches, the raster-based approach suggests that the rest of Richmond also has substantial heat health risk (light red colour in Figure 5 Top), while the vector-based approach suggests it does not (white colour in Figure 5 Bottom).

Also, by comparing the heat risk estimation from two approaches to the exposure and vulnerability data (Table 2), the heat risk from raster-based approach better stratified the data than vector-based result. The ranges of exposure and vulnerability data from raster-based approach were much wider than the results from vector-based model.

### 3.2. Multi-Scale Hotspot Analysis

**Figure 6:**
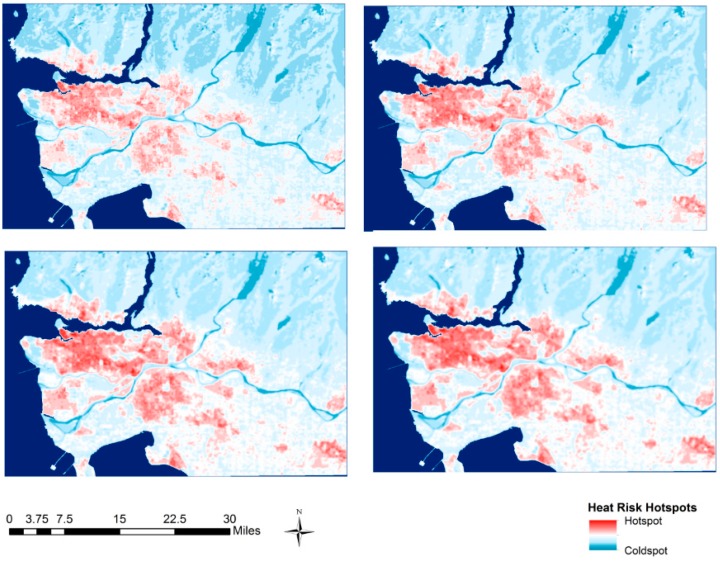
Comparison of heat risk hotspot maps produced with lag distances ranging from one pixel (**Top Left**) to four pixels (**Bottom Right**). Central Burnaby and East Vancouver (labelled in Figure 2) is known to have relatively high social vulnerability [63] and heat exposure [30], and contains the large heat risk hotspot in the study area.

Figure 6 shows the multi-scale hotspot results using lag distances between one and four pixels, corresponding to 180 m and 540 m. All lag distances result in maps that visualize the broad spatial trends in the data, locating areas with high heat exposure and high social vulnerability [31,63] such as low-income neighborhoods in East Vancouver and Central/South Burnaby that are likely associated with relatively higher heat risk [36]. Hotspots produced with the single-pixel lag distance are relatively more isolated and show greater local variability, while the four-pixel lag distance produces a smoother map with less local variability and clearer broad spatial patterns. This smoothing effect is inherent to the G_i_ index and other spatial filtering methods.

**Table 2:** Comparison of heat risks from raster-based and vector-based model. Each type of heat risk was evaluated with the mean value of exposure and vulnerability variables.

	Risk Category	Mean LST	Mean Seniors	Mean Infants	People in Old Houses	People in High Heat Risk Homes	Low Income Population	Low Education Population	People Living Alone	Unemployment Rate
1	Raster-based	292.4	7.2	0.8	3.1	1.3	1.3	11.4	2.1	2.1
	Vector-based	299.6	6.1	0.6	1.5	1.2	0.8	7.6	1.5	2.3
2	Raster-based	297.9	8.4	0.9	2.6	3.3	1.4	10.0	2.6	2.4
	Vector-based	305.1	57.1	6.1	22.0	18.3	10.2	77.7	17.5	3.9
3	Raster-based	301.8	20.6	2.0	7.6	4.8	3.0	27.9	5.3	3.1
	Vector-based	308.1	227.8	25.9	85.1	63.5	33.5	323.1	62.8	4.5
4	Raster-based	307.0	80.3	8.2	31.2	14.8	11.3	111.2	19.3	3.9
	Vector-based	309.7	496.4	61.0	215.8	136.5	86.6	745.4	140.8	6.6
5	Raster-based	311.3	412.4	50.6	180.2	95.2	69.6	639.0	112.0	5.4
	Vector-based	312.0	599.5	79.1	307.6	235.0	122.8	967.0	210.3	5.5
6	Raster-based	313.1	1002.3	130.1	487.3	630.7	263.5	1637.5	434.3	7.4
	Vector-based	312.7	969.0	128.0	538.5	773.1	292.2	1556.2	522.2	6.0
7	Raster-based	313.3	2343.0	283.5	1505.0	3601.6	1151.8	3578.1	2019.4	7.9
	Vector-based	313.6	1483.9	168.6	762.1	1419.7	510.5	2450.7	860.0	7.2
8	Raster-based	312.6	4871.9	654.5	4788.2	11951.1	4233.6	7317.8	6809.0	9.0
	Vector-based	314.2	2126.5	242.9	1347.5	2979.9	1155.6	3466.5	1705.6	8.2
9	Raster-based	313.9	6073.5	298.9	9284.7	14965.3	11314.6	12507.8	12110.7	14.8
	Vector-based	314.6	4360.7	634.0	5211.2	9453.2	4475.1	7183.6	5847.5	8.5

## 4. Discussion

### 4.1. Comparison of Resampling Approaches

The maps in Figure 3 and Figure 4 illustrate the importance of the MAUP issue, specifically in the context of MCA analysis. The raster-based approach was designed to retain the spatial resolution of the heat exposure data, which resulted not only in a description of heat health risk that included greater local variability, but also produced substantially different predictions of heat health risk for a large area in Richmond. While the raster-based approach avoids aggregation of temperature data into the dissemination area polygons, it implicitly assumes that the vulnerability data for the dissemination area in question are a valid representation of vulnerability in each raster cell covered by the area. Especially in dissemination areas that cover relatively large neighbourhoods, this assumption will necessarily generate some level of error in the pixel-level heat health risk estimates. The MAUP issue is thus not avoided, but rather changed in nature. No analysis has been conducted to assess which of the two maps provide the best description of the actual distribution of heat health risk in the area, but a comparison of the two maps with observed and geo-located heat-related mortality could provide such analysis. Such analysis would also provide additional information on the strength of the relationship between the heat health risk of an individual and the geographical location of that individual’s residence. While such relationship is an implicit assumption of geographically based heat exposure assessments (e.g., [16,43]), most adults spend the majority of the daytime at other locations, with uncertain and variable influence on their cumulative daily heat exposure.

### 4.2. Multi-Criteria Decision Analysis

We used equal weights to combine the eight vulnerability layers, and subsequently to combine the vulnerability and exposure layers to produce the risk layer, because no information exists from which a more appropriate weighting can be derived. While a strength of MCA is indeed the ability to provide different weighting of variables with different importance, the lack of both sufficient expert knowledge and quantitative information for calibration of weights and/or inclusion/exclusion of vulnerability variables is a limitation of both this and other similar studies [2,24,25,26]. Furthermore, there are indications that intra-urban variability in the temperature-mortality relationship can exist [14,16], in which case MCA weights should ideally be geographically variable. However, without extensive local calibration data such variability, and any similar variability in the vulnerability-mortality relationship, is not possible to include in the analysis. Future research should address ways to conduct such calibration, ideally using georeferenced mortality data from past extreme heat events when these are available and sufficiently extensive.

We used LST to quantify heat exposure because this is the most commonly used heat exposure variable in spatial heat health research [16,18,24,25,26], and because derivation of LST from publicly available Landsat data is relatively straightforward. Replication is thus possible for health geographers, public health scientists, urban planners and others who may have limited experience with processing of satellite data. Heat exposure measures based on air temperature or apparent temperature (a combination of air temperature and humidity) are likely more directly related to the effect heat has on human health [64,65], and are the norm in the non-spatial heat health literature (e.g., [12]), but such heat exposure measures are more difficult to map, subject to larger errors in their per-cell temperature estimates, and require extensive local calibration [31,66].

Despite possibilities for improvement, the results of the present analysis are sensible in the context of local heat-health studies [8,15], and represent a good assessment of the spatial distribution of heat health risk in the area. Importantly, the present analysis relies entirely on publicly available data, allowing straightforward replication elsewhere in Canada and beyond.

### 4.3. Hotspot Analysis

We used the multi-scale hotspot analysis to identify areas of elevated heat health risks at a range of spatial scales. Because the hotspot analysis is based on the regular grid provided by the raster approach it is not significantly influenced by the arbitrary boundaries of census dissemination areas and thus less subject to zoning issues related to MAUP [27]. The primary utility of this approach is that practitioners may benefit from identification of hotspots at a specific scale that suits their specific information needs. For example, at one scale regional health authorities may be able to identify priority municipalities to work with, while at another scale the municipalities may identify specific hotspots that can be targeted for heat mitigation measures such as urban greening or water fountains. While this provides substantial flexibility, it does require users to consciously and intelligently select spatial scales suitable for their specific needs.

## 5. Conclusions

Mortality caused by extreme heat is a global phenomenon expected to increase in severity as a result of global climate change. Spatial data on population vulnerability and heat exposure can be combined to map the health risk associated with extreme heat events. We illustrated a raster-based and a vector-based approach to combine census data and thermal satellite imagery in a multi-criteria analysis, and used the Getis-Ord G_i_ index to conduct a spatially smoothed hotspot analysis for the greater Vancouver area in Canada. The results illustrate that the raster-based approach reduces the potential modifiable areal unit problem, and produces more detailed maps of local spatial variability in heat health risk. The Getis-Ord G_i_ index with a range of lag distances allowed production of hotspot maps at a range of spatial scale as a useful tool for urban planners engaging in heat mitigation planning. The approach demonstrated in this study could increase the spatial specificity of heat risk predictions, which may improve the quality and cost-effectiveness of heat mitigation and emergency planning. Geocoded mortality data could potentially be used for local model calibration.
